# Recommendations for the urgent need to vaccinate school-aged and adolescent children against COVID-19 in the Asia–Pacific region

**DOI:** 10.1186/s41182-021-00365-5

**Published:** 2021-09-16

**Authors:** Jun Kobayashi, Rie Takeuchi, Fumiko Shibuya, Yuki Murata, Kenzo Takahashi

**Affiliations:** 1 Department of Global Health, Graduate School of Health Sciences, University of the Ryukyus, 207 Uehara, Nakagami-gun, Nishihara, Okinawa 903-0215 Japan; 2 Japanese Consortium of Global School Health Research, Okinawa, Japan; 3 Department of General Pediatrics, Tokyo Metropolitan Children’s Medical Center, Tokyo, Japan; 4 Graduate School of Public Health, Teikyo University, Tokyo, Japan

**Keywords:** COVID-19, Vaccine, Adolescent, School-aged children, Asia, Western Pacific region

## Abstract

We recommend urgent expansion of a vaccination program for adolescents and school-age children against SARS-CoV-2 infection in the Western Pacific region. Since July 2021, SARS-CoV-2 infections in children have increased rapidly in this region. As infection rates rise due to the SARS-CoV-2 B.1.617.2 (Delta) variant, current preventive strategies such as mask wearing and social distancing have controlled its spread effectively. Prolonged school closure is currently being promoted to suppress virus spread among children. However, the negative impact of prolonged school closure is significant. Although vaccination of children under 12 is still controversial, preparations must be made now for their vaccination.

## To the editor:

Since July 2021, cases of SARS-CoV-2 infection have increased rapidly in the Western Pacific region. According to the World Health Organization (WHO), reported cases in this region have been lower than those elsewhere from 2020 to June 2021 [1]. The weekly number of cases reported on June 28 in the Western Pacific region was 128,103, which was less than that in other regions: 991,946 cases in America, 544,679 in Europe, 612,933 in Southeast Asia, 245,930 in the Middle East, and 203,938 in Africa. This might be due not to the widespread use of vaccines but to cellular immunity, which might be acquired as an inborn characteristic by the inhabitants of the Asian region [2], or the thoroughness of hygienic behaviors such as hand washing and wearing of masks, and the success of measures to restrict human flow [3–5]. As of the end of June, vaccine coverage was 68% in Canada, 66% in the UK, 55% in Germany, and 54% in the USA, whereas only Singapore and China in the Western Pacific region were above 50%. The rate was 26% in Japan, a high-income country, 7.1% in the Philippines, a lower middle-income country, and only 3.7% in Vietnam [6]. However, since the beginning of July 2021, the rate of weekly increase in cases has risen rapidly, ranging from 10 to 35% [1]. This is thought to be due in large part to the influx of SARS-CoV-2 B.1.617.2 (Delta). The Delta variant is reported to be an extremely highly contagious strain [7] and has become the predominant strain in much of the region as in other regions [8]. The rate of positivity of the Delta strain is reported to be about 67% in screening tests performed from 26 July to 1 August in Japan. Its numbers continue to rise and are replacing those of other variants. In Tokyo, in particular, the rate has been about 80%, and the latest estimate is about 95% [9]. The effective reproduction number (Rt) was reported to exceed 1 in Tokyo in July and more than 2 in Okinawa at the beginning of August [10]. It can be inferred that this virus is easily spread despite the fact that nearly 100% of the people in Japan wear masks when going out.

## Increasing risk of infection among children

The influx of the highly infectious Delta strain has put unvaccinated children in the region at greater risk of infection. On August 14, 2021, 1902 children were reportedly hospitalized in the United States. The Delta variant is spreading rapidly, mainly among the unvaccinated population, and hospitalizations have skyrocketed in recent weeks, and that of children under 12 years of age has surged to a record high [11]. Although children in this age group are not eligible for vaccination, they are still vulnerable to the Delta variants. A similar trend has been observed in Japan, with a sharp increase in infections occurring among children. Vaccination of children over 12 years of age has been approved; however, due to priority to adult vaccination, many children over the age of 12 in Japan are not being vaccinated, putting the entire population at risk, including adolescents [12]. So far, there is no indication that the spread of Delta strains has increased the rate of severe cases and sequelae. However, there are numerous reports of sequelae occurring after SARS-CoV-2 infection has been cured in all age groups including in children [13, 14]. It is estimated that the disease is spreading to children in other Western Pacific countries as well as the United States and Japan.

## Negative effects of prolonged school closures

Once the increased risk of infection in children is recognized by policy makers and society, it is likely that there will be prolonged school closures in the Western Pacific region, the negative impact of which cannot be ignored [15]. Several studies reported that during the school closures, the time spent on physical activity decreased but sedentary time increased [16, 17], which may cause overweight in children [18]. In addition, school closure result in undernutrition in those children who depend on free meals in schools [19]. During the school closure period, i.e., the home-based learning period, children’s sleeping time became longer than that before school closure, bedtime was later, and wake-up time was much later than that before school closure [17], indicating that children’s life rhythm has been disturbed. Moreover, increased screen time due to home-based learning using internet materials was thought to have had some impact as well [16, 20], and even non-academic screen time also increased, which might affect children’s sleep [17]. Furthermore, children’s mental health has been affected, with increasing reports of behavioral problems, anxiety, depression, and stress [21, 22].

## Development of a pediatric SARS-CoV-2 vaccine

Development of a pediatric SARS-CoV-2 vaccine is still under way, and new related evidence is expected to emerge. As of August 20, 2021, evidence on mRNA and inactivated vaccines has been reported. As a potential pediatric COVID-19 vaccine, the mRNA-1273 SARS-CoV-2 vaccine has finished progression to a phase 2–3 study in adolescents (13–17 years) and has shown efficacy in preventing SARS-CoV-2 infection [23]. As for mRNA vaccines, WHO, the US-CDC and the Mayo Clinic recommend BNT162b2 SARS-CoV-2 vaccine for adolescents [24, 25]. In terms of vaccine safety and acceptability, according to the US-CDC data on BNT162b2 SARS-CoV-2 vaccine, younger vaccine recipients were more prone to experience common side effects than older recipients, even though the data have only been roughly summarized [26]. As of August 27, 2021, a Nature News explainer article, one of the fastest and most reliable sources of information on COVID-19, reported that a variety of vaccines are under clinical trial for children over 12 years, and up to July 20, 2021, when the article was published, the vaccines appear to be safe at least for adolescents [27]. In addition, due to the emergence of variants of concern, the role of adolescents and young adults in the pandemic is coming into the limelight. In short, at least adolescents may contribute to transmission in families and communities that can be hampered by vaccines for adolescents [28]. As available evidence for other vaccines, a double-blind, randomized, controlled trial of an inactivated SARS-CoV-2 vaccine named CoronaVac conducted in 3–17 years has been completed without serious adverse effects being reported [29]. Thus, considering the trade-offs of risks (non-safety events) and benefits (protection from COVID-19), the benefits of this vaccine outweigh the risks.

The recent data suggest that the vaccine is safe, but there are limitations regarding ethnic bias and sample size, so we hope that this trial will continue. As of August 5, 2021, the American Academy of Pediatrics requested the US Food and Drug Administration to work continuously to authorize safe and effective COVID-19 vaccines for children under the age 12 as soon as possible [30].

## Urgent need for vaccination of children in the region

Countries in the Asia–Pacific region should urgently implement vaccination against SARS-CoV-2 in adolescents to avoid prolonged school closures. We need to be prepared to start vaccinating school-age children under 12 years of age as soon as the safety of the vaccine is reported. We do not deny that the elderly are a priority group for vaccination, because their susceptibility to severe disease is indisputable. However, as of August 2021, when many countries have achieved higher vaccination rates for the elderly, there is no reason to refrain from vaccinating children except for the fact that vaccine safety in children has not yet been confirmed. It is not as easy to prevent the spread of the virus among children in schools, as it is among adults because of the high infectivity of the Delta strain. It is much more difficult for school children to maintain social distance and to limit conversations at meals than it is for adults. In addition, children can be assumed to be spreaders of the virus and can potentially infect the elderly. In low- and middle-income countries, many poor people, from children to the elderly, live in small rooms. Furthermore, the efficacy of the vaccine in preventing both infection by the Delta strain and severe disease is reported to decline with time after vaccination. [31, 32].

With consideration of the severity of COVID-19 in children, more attention needs to be paid to children with underlying diseases. More than 2000 deaths have already been reported in Brazil and more than 1500 in India. The reported problems are due not only to access to medical services, but also to obesity and air pollution caused by poverty [33]. Agarwal et al. reported two severe cases of obese adolescent females [34]. In Cook islands, Nauru, Niue, Samoa, Tonga and Tuvalu, more than 40% of adolescent are reported to be obese, and obese children have become a major public health challenge in many of Pacific Island countries [35, 36]. In the Asia–Pacific region, 4 billion people are exposed to air pollution, and the air pollution in the Western Pacific region in the metropolitan areas of China, Korea, Japan, and Vietnam cannot be ignored [37]. A cohort study in the United States in 2020 showed that asthma may not be a risk factor for COVID-19, and in Chengdu, China, asthma in children was reported to be improving as a result of the lockdown there [38, 39]. However, if the new Corona virus infections are controlled in the adult population and social activities increase, air pollution will once again become serious in the megacities of Asia. It will be necessary to observe whether this effect will impact the severity of the disease in children.

We believe that the creation of opportunities to inoculate both parents and children at the same time will be effective. In large companies, vaccinations are administered simultaneously in the workplace, but in smaller companies, it is necessary to get vaccinated individually. Flexible vacation time is not always recommended by companies for this reason, and in some cases, companies do not encourage their employees to leave the workplace for vaccinations. Vaccination hesitancy comes in stages, and not all people remain unvaccinated due to strong personal beliefs, but are influenced by the social mood [40]. Schools can be one important place to create a positive social mood to promote vaccination. The Asia–Pacific region is one of the most advanced regions in the world in implementing Health Promoting Schools, so schools already have experience in creating a healthy and positive social environment [41]. We have shown that health behaviors associated with infectious diseases can be effectively disseminated from children to the community [42, 43]. Schools will be able to inform not only children, but also their parents about COVID-19 control that includes vaccination promotion.

The WHO advisory group has already mentioned the future possibility of vaccinating children over the age of 12 because of the reported safety of the Pfizer vaccine. We expect that from now on, the safety of the Pfizer vaccine for children under 12 years and that of other companies’ products will be reported [44]. Based on current experiences in each country of the region, it is quite possible that there will be delays in the approval process for vaccination by the governments and delays in the supply of vaccines to the various countries and in the development of supply systems by local governments and school-level vaccination sites. In particular, low- and middle-income countries have limited resources and will need adequate time to prepare. The problem of vaccine refusal is expected to increase as parents become more sensitive to safety issues, especially when it is their children who are being vaccinated. Therefore, from now on, central and local governments should request the education sector to convey appropriate information through conversations between the health and education sectors. At the school level, it will be necessary to use the experience gained to date to not only to reinforce transmission in schools, but also to strengthen the partnership with parents and communities in the fight against the new corona variants. In addition, some Pacific Island countries have not strengthened other public health approaches because of long-term border closure measures, and thus, it will be necessary to incorporate vaccination of school children into international assistance programs from now on as we continue to attempt to control the COVID-19 pandemic.

## Data Availability

Not applicable. The manuscript does not contain any data.

## Abbreviations

COVID-19: Coronavirus disease 2019
WHO: World Health Organization
